# Psychometric evaluation of the *Bangla-Translated Rotter’s Internal-External Scale* through classical test theory and item response theory

**DOI:** 10.3389/fpsyg.2022.1023856

**Published:** 2022-11-11

**Authors:** Mushfiqul Anwar Siraji, Shamsul Haque

**Affiliations:** ^1^Department of Educational and Counselling Psychology, University of Dhaka, Dhaka, Bangladesh; ^2^Department of Psychology, Jeffrey Cheah School of Medicine and Health Sciences, Monash University Malaysia, Bandar Sunway, Malaysia

**Keywords:** locus of control, structural validity, classical test theory, item response theory, concurrent validity

## Abstract

There is no psychometric tool to assess locus of control for Bangla-speaking people. Hence, we attempted to translate the 23-item Rotter’s Internal-External scale into Bangla and validate it on Bangladeshi adult participants. In Study 1 (*N* = 300), we translated the items into Bangla and conducted an exploratory factor analysis, which gave a one-factor solution with 12 items. In Study 2, we conducted a validation study (*N* = 178) to accumulate evidence on the structural and concurrent validity of the 12-item scale. Structural validity assessed by confirmatory factor analysis yielded the best fit for the one-factor model with 11 items (CFI = 0.98, TLI = 0.97, RMSEA = 0.00). The scale’s significant correlations with Internal Control Index, which is also a measure of locus of control (*r* = −0.22, *p* < 0.01), Neuroticism (*r* = 0.21, *p* < 0.01) and Openness to Experience (*r* = −0.22, *p* < 0.01) demonstrated its satisfactory concurrent validity. Reliability coefficient of this 11-item scale was satisfactory (McDonald’s Omega total = 0.72). The item quality was assessed on the combined samples of Study 1 & 2 (*N* = 478) using the item response theory (IRT), which showed that the scale covered a sizable range of the underlying locus of control with items varying in difficulty (−1.09–2.79). Item discrimination analysis indicated sufficient discriminating power of the items (0.49–2.21). The test information curve showed the scale’s adequate ability to discriminate between external and internal locus of control. IRT analysis also indicated satisfactory marginal reliability for the scale (0.72). These psychometric properties suggest the usability of the Bangla version of Rotter’s Internal-External scale.

## Introduction

Locus of control (LoC) is the individual’s belief regarding the contingency of the reinforcement on their internal qualities and behavior vs. other external attributes like chance or fate (Rotter, 1966). The LoC can be viewed as a bipolar continuum where internal and external are two extremities, indicating an individual’s disposition on the reinforcement expectancy. Individuals with internal LoC believe that the reinforcement and fundamental control over the event’s outcomes are contingent on their ability, behavior and efforts. However, for individuals with external LoC, the fundamental sense of agency of life and reinforcement are bestowed on the attributes like fate, luck, change or other powerful entities (Rotter, 1966; Rotter et al., 1972; Marsh and Richards, 1987).

Rotter (Rotter, 1966) developed the Internal-External (I-E) scale based on the social learning theory (Rotter, 1954, 1955, 1960) to measure individual differences in reinforcement expectancy. Social learning theory assumes if a reinforcement is not contingent upon people’s behavior, the reinforcement will not strengthen that particular behavior (Rotter, 1966). This expectation of reinforcement to be contingent upon behavior may affect a broad range of behaviors in different domains including academic achievement (Findley and Cooper, 1983; Karaman et al., 2018), health (Jacobs-Lawson et al., 2011), professional competence (Witt, 1988; Mantesso et al., 2008; Smidt et al., 2018; Diotaiuti et al., 2021) and consumer behavior (Lee et al., 2018; Rodriguez-Ricardo et al., 2019). Internal LoC is attributed to better health care management, self-assessment (Pourhoseinzadeh et al., 2017) and academic success (Karaman et al., 2018). However, external LoC is associated with increased depression, anxiety, stress (Kurtovic et al., 2018), and different personality factors, such as high neuroticism (Horner, 1996) and low openness to experience (Sherman et al., 1973; Kobasa et al., 1982; Taylor, 1983). Assessment of LoC is beneficial in different therapeutic interventions (Baker, 1979; Delsignore and Schnyder, 2007). Individuals with internal LoC are more receptive to the information (Cavaiola and Strohmetz, 2009), more resilient and hopeful than individuals with external LoC, thus facilitating the favorable outcome in the psychotherapy (Foon, 1987). LoC also facilitates the “Transactional Analysis” based counseling process by indicating an individual’s predominant ego-states (Loffredo, 1998). Internal LoC is associated with the “Adult” ego state and External LoC is associated with the “Adapted Child” ego state (Loffredo, 1998).

Rotter’s Internal-External (I-E) scale (Rotter, 1966) was published in 1966 and is the most widely used scale to measure the LoC. It has been adapted across different countries including Poland (Tobacyk, 1978), Netherlands (Andriessen and Van Cadsand, 1983), Australia (Watson, 1981), Brazil (Nagelschmidt and Jakob, 1977), and Sri Lanka (Niles, 1981). Smith et al. (1995) listed 58 countries that used either an adapted or a translated version of this scale to measure LoC. However, the scale was originally developed and validated in the US, which is an individualist culture. Members of individualist culture emphasize highly on personal life choices, whereas members of collectivist cultures emphasize the membership of groups (Hofstede, 1980).

Smith et al. (1995) identified some fundamental problems of using Rotter’s I-E scale in collectivist cultures, for example ‘modesty bias’, whereby individuals’ responses might represent the group’s opinion instead of the individual preference. In addition, values parallel to LoC, such as ‘mastery over the environment’ and ‘harmony with the environment’ are differentially endorsed by members of different cultures (Schwartz, 1990, 1992). This indicates the cultural susceptibility of the construct LoC, which may contribute to different latent structures found across various cultures. Although Rotter (Rotter, 1966) mentioned his scale to have one general factor with several other less essential factors and suggested the structure as unidimensional, studies in the United States (Mirels, 1970; Joe and Jahn, 1973) and other countries (Tobacyk, 1978; Niles, 1981; Marsh and Richards, 1987; Tyler et al., 1989) have established the multidimensional nature of this scale. Marsh and Richards (1987) summarized 20 studies that analyzed the latent structure of this scale and found that the number of possible interpretable factors ranged between 2 to 6 with two recurring factors: political control and personal control. These two factors in Rotter’s I-E Scale were first reported in the work of Mirels (1970). Items clustered under “personal control” stemmed from the individual’s inclination to prefer personal ability and hard work over luck. The “Political control” focuses on the individual’s disposition regarding their ability to control the political and world affairs as a part of the social system.

Smith et al. (1995) analyzed a databank of 9,140 responses to the Rotter’s I-E Scale (Rotter, 1966) to identify its latent structure. Respondents were business organization employees from 43 countries. They reported three recurring interpretable factors: “personal-political,” “individual-social” and “luck.” A trend of fatalism about political events and a high preference for luck among the included Asian nations was also observed in their study. Due to such cultural susceptibility, it would be inappropriate to use Rotter’s I-E scale without proper psychometric calibration in any Asian country.

There is a dearth of psychometrically valid scales in Bangladesh to assess LoC in a culturally sensitive way. We aim to fill this gap, by conducting two studies to culturally adapt and validate Rotter’s I-E scale. Our first objective was to translate Rotter’s I-E scale in Bangla and explore its latent structure (Study 1). The second objective was to validate the latent structure obtained in study-1 and gather concurrent validity evidence of Bangla Rotter’s I-E Scale (Study 2). The third objective was to assess the item quality of Bangla Rotter’s I-E scale using Item Response Theory - IRT (Study 2). For concurrent validity, we used the Internal Control Index (ICI)- a measure of locus of control (Duttweiler, 1984) and two subscales of the Big Five Inventory: neuroticism and openness to experience (John et al., 1991, 2008; Muhammad et al., 2011). ICI yields a high score for people with internal LoC whereas Rotter’s I-E yields a high score for external LoC. Thus, we predicted a negative correlation between ICI and Rotter’s I-E scores. Additionally, previous research has demonstrated the association of LoC with neuroticism and openness to experience. External LoC is associated with high neuroticism (Horner, 1996) and low openness to experience (Sherman et al., 1973; Kobasa et al., 1982; Tyler et al., 1989). Thus, we predicted a positive correlation of Rotter’s I-E scores with neuroticism and a negative correlation with openness to experience.

**Figure 1:**
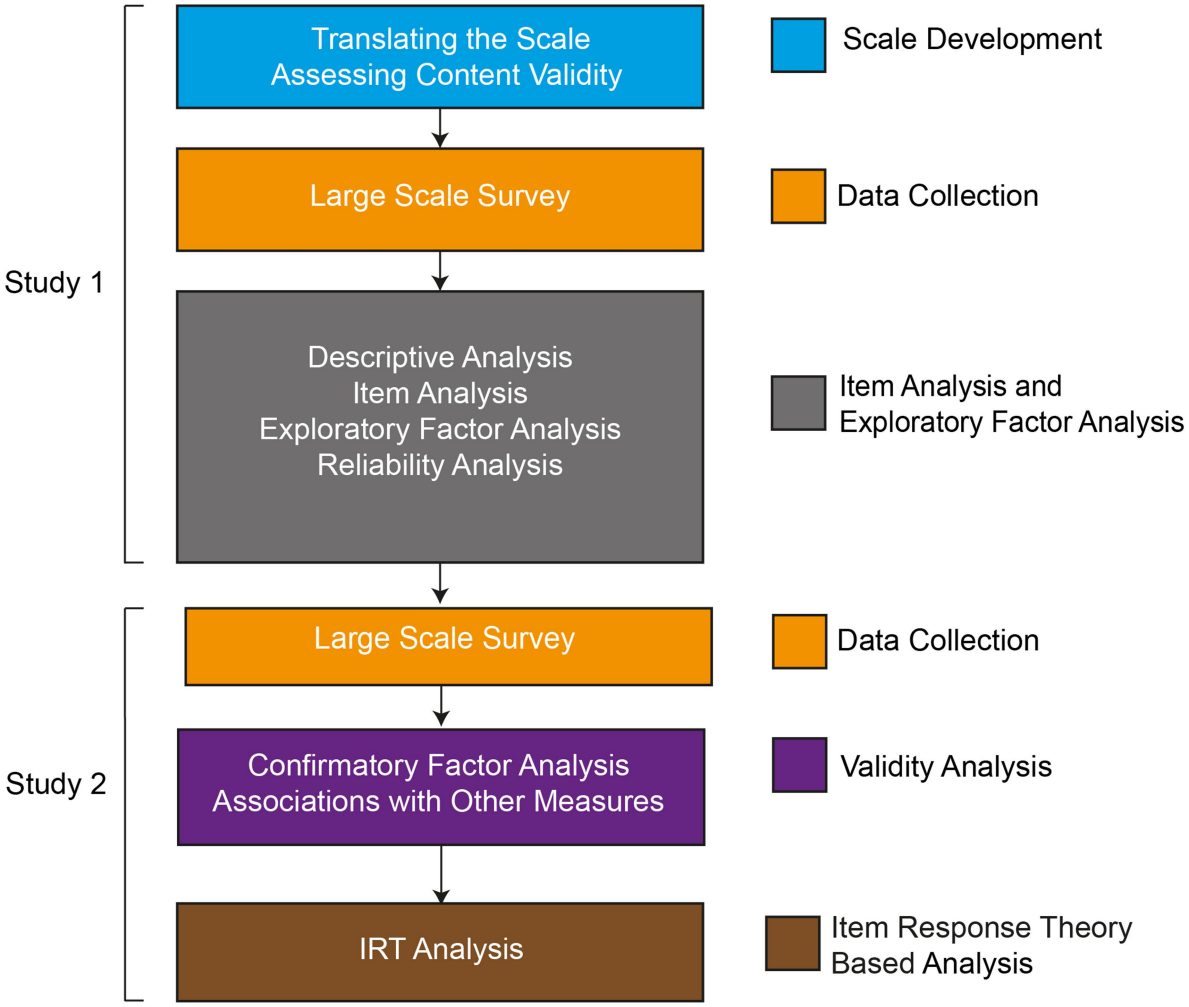
Flow chart of translation and psychometric evaluation of Bangla Rotter’s I-E scale.

To address the third objective, we assessed the item quality using IRT-based analysis on the combined sample of study 1 and 2. Most of the validation studies investigating the latent structure of Rotter’s I-E scale have employed Classical Test Theory (CTT) based analysis. CTT uses a set of concepts (unobservable construct, observed score, reliability) and provides information on the whole scale (DeVellis, 2006). CTT attributes the observed scores obtained on a scale to the unobservable construct of interest and possible measurement errors. The reliability coefficients indicate how closely the observed score reflects the unobservable construct (DeVellis, 2006). To strengthen the CTT-based analysis, we employed the IRT-based analysis to assess the item discrimination and difficulty of the Bangla Rotter’s I-E scale. Item discrimination is items’ ability to discriminate among the respondents across the latent construct continuum (Kazemi and Kajonius, 2021), and item difficulty indicates the level of latent construct a respondent requires to attain a 50% chance to score toward the desired direction for a particular item (Kazemi and Kajonius, 2021). Figure 1 depicts the steps we have followed in our psychometric evaluation of Bangla Rotter’s I-E Scale.

## Study-1: Translations and exploratory factor analysis

First, we translated 23 item pairs of the entire I-E scale from English to Bangla in a culturally meaningful way. Second, we assessed the content validity of the Bangla-translated scale. Third, we conducted an exploratory factor analysis to understand the latent structure of the scale.

### Method

#### Participants

We conducted a large-scale online survey to gather data for this study. Any Bangladeshi citizen who was >18 years of age and able to read and write Bangla was eligible to participate in this survey. In study-1, 312 Bangladeshi adults completed the survey. However, we excluded 12 participants, as their data were incomplete, leaving data for 300 participants for further processing. To explore the initial factor structure, a sample of 250–300 is recommended (Comrey and Lee, 1992; Schönbrodt and Perugini, 2013). Our sample size fulfilled this recommendation. Out of 300 participants, 66% were females, ranging in age from 21 to 52 years (29.20 ± 4.92), and 34% males, ranging in age from 21 to 45 years (32.39 ± 4.17). The average length of year of schooling for females was 15.28 ± 2.09 years, and for males, it was 16.71 ± 0.94 years. Nearly three-quarter (71%) of the participants were married.

#### Material

##### Rotter’s internal-external scale

Rotter’s Internal-External (I-E) scale has 23 item pairs in a forced-choice format and six additional filler pairs. Each pair contains one statement focusing on internal LoC and another focusing on external LoC [example: item 1. (a) Children get into trouble because their parents punish them too much; (b) The trouble with most children nowadays is that their parents are too easy with them]. The total score ranges from 0 to 23, with a higher score indicating higher external LoC. Internal consistency Kuder–Richardson coefficient was 0.69 in the original scale among the nationally representative sample of United States of America (Franklin, 1963).

##### Bangla Rotter’s internal-external scale

We followed International Test Commission (ITC) Guidelines (Bartram et al., 2018) to translate and adapt Rotter’s I-E scale. Two bilingual researchers (PhD in Psychology) from Bangladesh translated the original English version to Bangla. Two translated versions were then judged and synthesized by the authors. Subsequently, two other bilingual researchers (One PhD, one MS in Psychology) from Bangladesh back-translated the Bangla scale into English with no knowledge of the original work. The authors synthesized the two back-translations, compared it with the original scale and made necessary amendments.

#### Data collection

The project received ethics clearance from Monash University Human Research Ethics Committee (Project ID: 30638). A quantitative cross-sectional fully anonymous online survey was conducted. Participants were invited *via* email and social media (i.e., LinkedIn, Twitter, Facebook) with the attachment of an Explanatory Statement. It was mentioned in the explanatory statement that their participation was voluntary and that they could withdraw from participation anytime without being penalized. If the participants expressed happiness with the Explanatory Statement, a survey link was sent to them. At the beginning of the survey, their consent was recorded digitally. The survey took around 15 to 20 min for which they were not compensated. We collected the survey data between 17 January 2022 and 3 March 2022.

#### Analytic strategy

We used R (version 4.1.0) (Team, 2021), including several R packages (Chalmers, 2012; Rosseel, 2012; Revelle, 2021; Siraji, 2021; Barnier et al., 2022), for our analyses. Since Rotter’s I-E scale used a dichotomous forced-choice response and both univariate (Table 1) and multivariate normality assumptions were violated, we performed the exploratory factor analysis using a tetrachoric correlation matrix which was more robust to those violations (Watkins, 2020). We employed weighted least squares (WLS) as a factor extraction method to examine the latent structure of the scale. WLS is more robust toward violation of normality assumptions (Fabrigar et al., 1999). An orthogonal rotation technique: varimax was chosen following the literature investigating the latent structure of Rotter’s I-E scale (Mirels, 1970; Joe and Jahn, 1973; Tobacyk, 1978). Before the EFA, necessary assumptions, including sample adequacy, and quality of correlation matrix were assessed. As the commonalities for each item found in the previous studies were not higher than 0.70 (Mirels, 1970; Joe and Jahn, 1973; Tobacyk, 1978), we relied on the Scree-plot rather than the Kaiser criterion of eigenvalues greater than one (Stevens, 2009). We supplemented the scree plot (Cattell, 1966) with Horn’s parallel analysis (Horn, 1965), Minimum average partials method (MAP) (Velicer, 1976), and Hull method (Lorenzo-Seva et al., 2011). We compared the root mean square of the residual (RMSR) values obtained for the solutions to determine the best factor structure. RMSR ≤0.08 was preferred (Brown, 2015). Additionally, to identify the simple structure, we followed the following guidelines recommended by psychometricians (i) no factors with fewer than three items (ii) no factors with a factor loading <0.3 (iii) no items with cross-loading greater than 0.3 across factors (Child, 2006; Mulaik, 2009; Bandalos and Finney, 2019; Watkins, 2020).

**Table 1:** Descriptive statistics of 23 items of Bangla Rotter’s I-E scale (Study 1, *N* = 300).

Items	Mean	SD	Skew	Kurtosis	Normality	Corrected item-total correlation
Item 02	0.17	0.37	1.78	1.17	0.45^*^	0.24
Item 03	0.87	0.34	−2.15	2.62	0.40^*^	0.13
Item 04	0.43	0.50	0.30	−1.92	0.63^*^	0.28
Item 05	0.14	0.34	2.10	2.44	0.41^*^	0.25
Item 06	0.32	0.47	0.75	−1.44	0.59^*^	0.08
Item 07	0.85	0.35	−1.99	1.96	0.42^*^	0.23
Item 09	0.29	0.45	0.94	−1.12	0.57^*^	0.41
Item 10	0.08	0.28	3.00	7.02	0.31^*^	0.29
Item 11	0.53	0.50	−0.11	−2.00	0.64^*^	0.44
Item 12	0.49	0.50	0.03	−2.01	0.64^*^	0.29
Item 13	0.55	0.50	−0.20	−1.97	0.63^*^	0.39
Item 15	0.54	0.50	−0.17	−1.98	0.63^*^	0.47
Item 16	0.29	0.45	0.92	−1.16	0.57^*^	0.39
Item 17	0.81	0.40	−1.55	0.39	0.48^*^	0.17
Item 18	0.80	0.40	−1.52	0.31	0.49^*^	0.50
Item 20	0.52	0.50	−0.07	−2.00	0.64^*^	0.22
Item 21	0.22	0.41	1.37	−0.13	0.51^*^	0.26
Item 22	0.26	0.44	1.09	−0.82	0.55^*^	0.24
Item 23	0.09	0.29	2.78	5.76	0.33^*^	0.35
Item 25	0.62	0.49	−0.51	−1.75	0.61^*^	0.53
Item 26	0.72	0.45	−0.98	−1.05	0.56^*^	0.20
Item 28	0.20	0.40	1.47	0.15	0.49^*^	0.29
Item 29	0.45	0.50	0.19	−1.97	0.63^*^	0.22

* *p* < 0.001.

### Results and discussion

#### Content validity

We gave the Bangla-translated Rotter’s I-E scale to eight mental health professionals in Bangladesh. They independently evaluated the relevance of the items (23 items) using a 4-point Likert type scale (1: not at all relevant, 2: slightly relevant, 3: quite Relevant, 4: Highly Relevant). We estimated the item-level content validity (I-CVI) and scale-level content validity index (S-CVI). Any item with an I-CVI score higher than 0.83 indicates an adequate content validity (Lynn, 1986; Polit et al., 2007). Two items were below the cut-off value thus we readjusted the translation and the experts judged them again. After adjustment, I-CVI scores of all items were acceptable. The S-CVI for the total scale was 0.94, estimated using the average method and indicated satisfactory content validity (Lynn, 1986; Polit et al., 2007).

#### Sampling adequacy

Sampling adequacy for exploratory factor analysis was investigated by Kaiser-Meyer-Olkin (KMO) measures of sampling adequacy (Kaiser, 1974). The overall KMO value for 23 items was 0.68, which was above the cut-off value of 0.50, indicating an adequate sample (Hutcheson, 1999).

#### Descriptive statistics and item analysis

Table 1 presents univariate descriptive statistics for the 23 items. Most of the items are skewed with high kurtosis values. The Shapiro–Wilk test of normality (Shapiro and Wilk, 1965) indicated all the items violated normality assumptions. Multivariate normality assumptions were investigated by Mardia’s test (Mardia, 1970). Multivariate skew = 89.25 (*p* < 0.001) and multivariate kurtosis = 582.32 (*p* < 0.001) indicated the violation of multivariate normality assumptions. Due to the violation of univariate and multivariate normality assumptions and the dichotomous force choice response option, tetrachoric correlation over Pearson’s correlation was chosen (Watkins, 2020).

**Figure 2:**
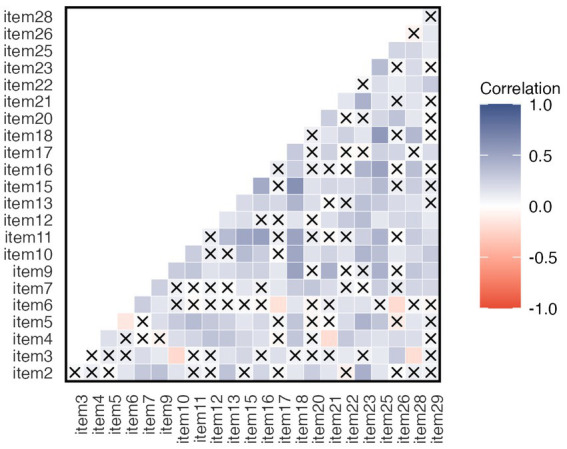
Inter-item tetrachoric correlation coefficients for the 23-item Rotter I-E Scale. “x” indicates non-significant correlations (*p* > 0.05). Inter-item correlation ranged between −0.22 and 0.62. 15.42% correlations were higher than |0.30|.

Figure 2; Supplementary Table S1 depict the inter-item correlation coefficients. Bartlett’s test of sphericity (Bartlett, 1954), *χ*^2^ (253) = 715.08, *p* < 0.001 indicated the correlations between items are adequate for the EFA. However, only 15.42% of the inter-item correlation coefficients were greater than |0.30| in the obtained matrix. The corrected item-total correlations ranged between 0.08 and 0.53 (Table 1). Such a low to moderate item-total correlation was also evident in the original scale, ranging between 0.11 and 0.48 (Rotter, 1966). As such, all items were retained.

#### Exploratory factor analysis

**Figure 3:**
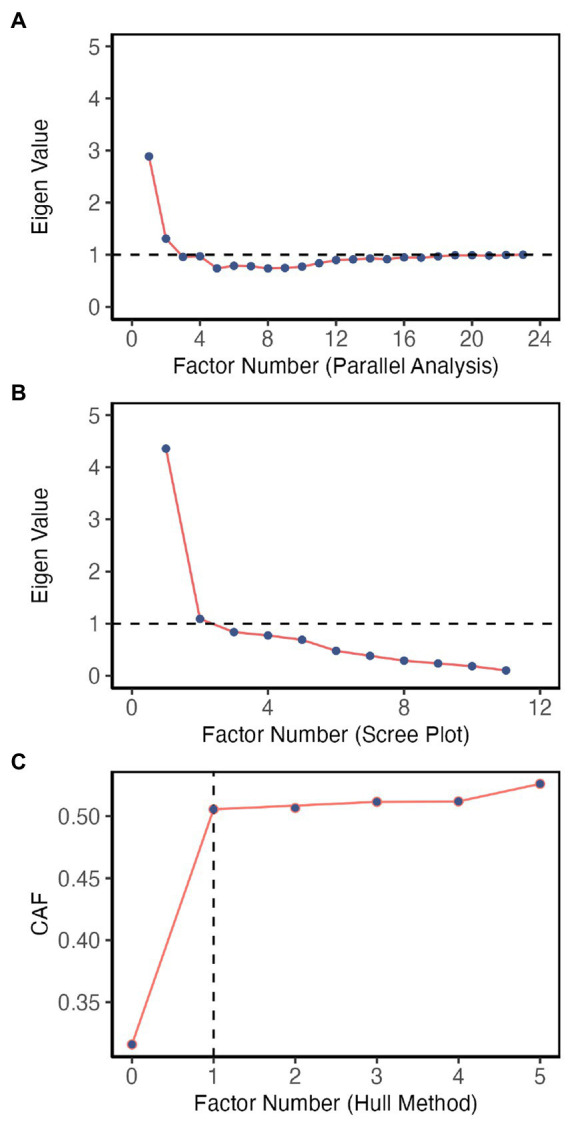
Factor identification methods used in exploratory factor analysis. **(A)** Parallel analysis indicates a two-factor solution. **(B)** Scree plot indicates a two-factor solution. **(C)** Hull method indicates the one-factor solution.

Horn’s parallel analysis (Horn, 1965), like Monte Carlo studies, draws several sets of random data sets with a sample size equal to the original data set and compares the mean eigenvalues among the simulated and original data sets to retain optimal factors. Parallel analysis is more immune to the normality assumptions violation (Garrido et al., 2013). In our data set, parallel analysis with 500 iterations indicated a 2-factor solution (Figure 3A). The scree plot (Figure 3B) suggested a two-factor solution. The minimum average partial (MAP) method expects the average squared off-diagonal values of the calculated partial correlation matrix to be minimum when the correct number of factors are extracted (Velicer, 1976). In our data set, these values reached the minimum after extracting the first factor, indicating a one-factor solution (Supplementary Table S2). The more contemporary “hull method” tries to find an optimal number of factors to balance model fit and the number of parameters (Lorenzo-Seva et al., 2011). This extraction method also supported a one-factor model (Figure 3C). As a result, we tested both one-factor and two-factor solutions.

The initial two-factor solution with all 23 items showed a lack of fit in terms of RMSR value (RMSR = 0.11), presence of cross-loading items (item 09 and 25) and items with poor factor loading (<0.30; item 06, 22 and 29). We discarded these items and ran another EFA with the remaining 18 items. This iteration of EFA also appeared as a misfit in terms of poor factor loading item (item 12). Another five rounds of EFA were conducted by gradually identifying problematic items and discarding them from the model. Finally, a two-factor EFA solution with 14 items was accepted with RMSR = 0.08, no loading smaller than 0.30 and no cross-loading greater than 0.30. The first factor retained nine items, and the second factor retained five items. The first factor explained only 20.5% of the total variance and the second factor explained only 9.6%. Marsh and Richards (Marsh and Richards, 1987) also reported such low explained variance by the factors while summarizing twenty explanatory factor analyses results on Rotter’s I-E scale. It was observed that the explained variance by the first factor ranged between 7 and 20%, and the second factor ranged between 7 and 10%. Table 2 summarizes the factor loadings of each retained item. The factor analysis revealed that our data supported congeneric model as the factor loadings of all items are different (Novick and Lewis, 1967; Graham, 2006). Subsequently, to get a better reliability estimate, we calculated McDonald’s *ω_t_* coefficient which follows the congeneric model (Novick and Lewis, 1967; Graham, 2006). The internal consistency of McDonald’s *ω_t_* coefficient for the first and second factors was 0.64 and 0.39, respectively. Both the factors suffered from poor reliability (Nájera Catalán, 2019).

**Table 2:** Factor loadings of two-factor solution from study 1 (*N* = 300).

Items	F1*	F2*
Item 18	0.78	
Item 11	0.75	
Item 15	0.65	
Item 16	0.56	
Item 10	0.47	
Item 05	0.45	
Item 13	0.44	
Item 28	0.42	
Item 04	0.38	
Item 20		0.64
Item 7		0.51
Item 21		0.44
Item 02		0.37
Item 26		0.33
% of Variance	0.20	0.10
McDonald’s *ω_t_*	0.64	0.39

* Only loadings higher than 0.30 is reported.

Next, we fit a one-factor solution, and after four rounds of identifying and excluding the problematic items, a simple structure with one factor was obtained with 12 items explaining 32% of the total variance (Table 3). The RMSR value was 0.09, slightly above the cut-off value (0.08). The internal consistency coefficient McDonald’s *ω*_t_ = 0.70 which was satisfactory (Nájera Catalán, 2019).

**Table 3:** Standardized factor loadings of one-factor solution from study 1 (EFA, *N* = 300).

Items	Study 1 EFA loadings*
Item 04	0.33
Item 05	0.45
Item 09	0.48
Item 10	0.53
Item 11	0.69
Item 13	0.45
Item 15	0.64
Item 16	0.61
Item 18	0.82
Item 23	0.48
item25	0.69
Item 28	0.44
% of Variance	32%
McDonald’s *ω_t_*	0.70

* Only loadings higher than 0.30 is reported.

The obtained one-factor solution retained all items of the first factor obtained in the two-factor solution. These items stemmed from the beliefs on the personal ability and effort versus external luck in successful personal goal achievement [e.g., Item 5: (a) The idea that teachers are unfair to students is nonsense; (b) Most students do not realize the extent to which their grades are influenced by accidental happenings]. Such a factor in Rotter’s I-E scale is supported in the literature (Mirels, 1970; Joe and Jahn, 1973; Tobacyk, 1978). The one-factor solution obtained in our study contained all nine items of the “personal control” factor found by Mirels (1970) with additional three items (items 04, 09, 13). These additional three items also focused on “personal control” [e.g., Item 09: (a) I have often found that what is going to happen will happen; (b) Trusting to fate has never turned out as well for me as making a decision to take a definite course of action]. However, the “political control” factor (Mirels, 1970; Tobacyk, 1978) reflecting the beliefs on people’s influence over political events was not evident in our sample. Items belonging to the second factor of the two-factor model in our study stemmed from the beliefs on interpersonal relationships [e.g., item 07: (a) No matter how hard you try some people just do not like you; (b) People who cannot get others to like them do not understand how to get along with others], and luck [e.g., item 21: (a) It is hard to know whether or not a person really likes you; (b) How many friends you have depends upon how nice a person you are]. The existence of a factor related to luck in Asian nations was also reported by Smith et al. (1995). However, in our sample the second factor was less interpretable in terms of a common theme and showed low internal consistency (McDonald’s *ω_t_* = 0.39). Thus, we retained the one-factor model, which had better reliability estimates, better RMSR value and meaningful interpretation.

## Study-2: Confirmatory factor analysis and concurrent validity of Bangla Rotter’s I-E scale

First, we confirmed the latent factor structure of Bangla Rotter’s I-E scale obtained in study-1 by confirmatory factor analysis thus providing structural validity and estimated the scale’s reliability. Second, we gathered evidence of concurrent validity for our adapted scale (Furr, 2014). Third, we gathered item difficulty and discrimination information on the adapted scale using the Item Response Theory (IRT) on the combined sample of studies 1 and 2.

### Method

#### Participants

We conducted another large-scale online survey to gather data for study-2. The eligibility criteria were same as study-1. In study-2, 178 Bangladeshi adults participated. There was no missing or incomplete data. Seventy-two percent 72% (129) of the participants were females, ranging in age from 21 to 53 years (29.20 ± 4.85), and 28% (49) were males, ranging in age from 26 to 44 years (33.30 ± 3.82). Seventy-nine percent of the participants were married. The average length of education years for males was 16.84 ± 0.37 years and for females was 15.14 ± 2.14 years. For estimating the sample size for the confirmatory factor analysis, we followed the N: q rule (Bentler and Chou, 1987; Jackson, 2003; Worthington and Whittaker, 2006; Kline, 2015), where 10 participants per item are required to earn the trustworthiness of the result. Our sample size exceeded the requirement as we had 12 items.

#### Materials

##### Bangla Rotter’s I-E scale

To confirm the latent structure of Bangla Rotter’s I-E Scale, we used the one-factor solution with 12 items obtained in Study-1.

##### Internal control index (ICI)

The ICI is a 28-items 5-point scale to measure a person’s LoC (Duttweiler, 1984). The items were translated into Bangla using the standard forward-backward translation procedure and the judgment of an expert panel. The internal consistency coefficient McDonald’s ω_t_ obtained in our sample was 0.86.

##### Bangla Big five inventory (BBFI)

We measured neuroticism and openness to experience by two subscales of BFI (John et al., 1991, 2008). We used the adapted Bangla BFI (Muhammad et al., 2011). The neuroticism subscale measures the extent to which an individual is affectively unstable, anxious and worried (Horner, 1996). It has eight items (3 reversed items). The openness subscale has ten items (2 reversed items) and measures an individual’s susceptibility to aesthetics, ideas, values and flexibility (Costa and McCrae, 1992). Each item was scored on a five-point Likert scale. The internal consistency coefficient McDonald’s ω_t_ for neuroticism and openness to experience obtained in our sample was 0.77 and 0.69, respectively.

#### Data collection

Ethics clearance for this study was obtained together with Study 1. Data was collected in a quantitative cross-sectional approach *via* a fully anonymous online survey. Participants were invited *via* email, and social media (i.e., LinkedIn, Twitter, Facebook) along with explanatory statements and upon their expressed interest, a survey link was sent to them. Once the participants voluntarily agreed to participate, their consent was recorded digitally. It was clearly explained to the participants that they could withdraw from participation anytime without being penalized. Completing the online survey took approx. 20 to 25 min and was not compensated. We collected the survey data between 15 April and 20 July 2022.

#### Analytic strategy

We used the ‘Lavaan’ (Rosseel, 2012) package in RStudio to conduct the categorical confirmatory factor analysis with Weighted Least Square with mean and variance adjusted (WLSMV) estimator as our response data was dichotomous (Brown, 2015). To estimate the model fit we adhered to commonly used model fit benchmarks of Hu and Bentler (Lt and Bentler, 1999): (i) the comparative fit index (CFI) and the Tucker Lewis index (TLI; CFI/TLI ranging between 90–95 and above) (ii) the root mean square error of approximation (RMSEA; close to 0.06 or below), (iii) the standardized root mean square (SRMR; close to 0.08 or below) to estimate the model fit. We have also estimated the *χ*^2^ test statistics for the fitted model. However, *χ*^2^ test statistics is sensitive to sample size (Brown, 2015). And, for categorical data SRMR also performs poorly (Yu, 2002). As such more importance was given to CFI, TLI and RMSEA fit indices.

We also gathered concurrent validity evidence from the correlational analysis between Bangla Rotter’s I-E scale and ICI (Duttweiler, 1984), neuroticism, and openness to experience (Muhammad et al., 2011). Lastly, we sought to Item response theory (IRT) to gather information on item difficulty and item discrimination of the Bangla Rotter I-E scale. IRT judges an item’s quality by providing item information (difficulty & discrimination) in the light of participants’ trait level (*θ*). In IRT-based analysis, our aim was only to assess the quality of the items retained in the CFA model. We followed the guidelines of Baker and Kim (2017) and Hambleton et al. (1991) to assess the quality of the items and categorize them. Item discrimination range used was 0.5≤ item discrimination ≥2.0, and item difficulty range used was-3.0≤ item difficulty ≥3.0. Item discrimination categorization was none = zero, very low = 0.01–0.34, low = 0.35–0.64, moderate = 0.65–1.34, high = 1.35–1.69, very high >1.70, and item difficulty categorization was very easy <−2, easy = −2 to −0.50, medium = −0.51 to 0.50, hard = 0.51–2, and very hard >2.

### Results and discussion

#### Confirmatory factor analysis and reliability estimation

**Figure 4:**
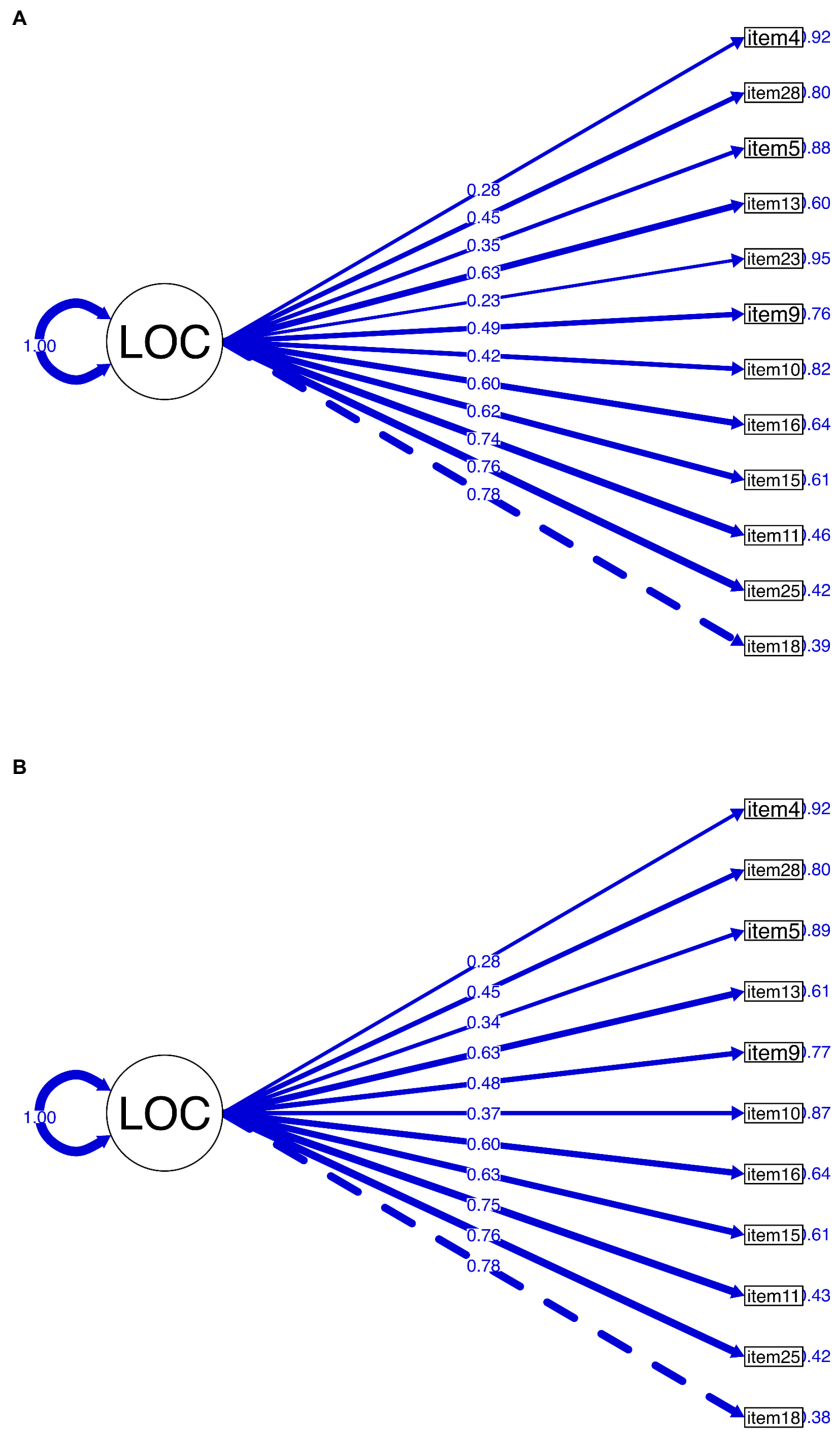
Confirmatory factor analysis on the one-factor model of Bangla Rotter’s I-E scale. **(A)** One-factor model with 12 items, **(B)** one-factor model with 11 items.

Table 4 summarizes the fit indices of our fitted models. One factor model with 12 items failed to attain an absolute fit estimated by the chi-square test (*χ*^2^ [54] = 83.84, *p* < 0.05). Another absolute fit index SRMR was also higher (0.12) than the general guideline. However, comparative fit indices (CFI = 0.92, TLI = 0.91) and parsimony index (RMSEA = 0.04) for the one-factor model with 12 items indicated acceptable fit. However, two items (item 23, item 04) loaded poorly (Table 5). By discarding one item with the poorest factor loading (item 23) our model attained the best fit (CFI = 0.98, TLI = 0.97, RMSEA = 0.00). SRMR value (0.10) was also closer to the suggested guideline (0.08). Thus, we accepted the later model (11-item model). The accepted 11 items are provided in Supplementary File S1. The internal consistency reliability coefficients McDonald’s ω_t_ for both models were 0.72. Figure 4 depicts both models.

**Table 4:** Summary of fit indices of the Bangla Rotter’s I-E scale (*N* = 178).

Model	*χ* ^2^	*df*	CFI	TLI	RMSEA 90% CI	SRMR	McDonald’s *ω_t_*
One factor (12 items)	83.84^*^	54	0.94	0.92	0.04 (0.03–0.08)	0.12	0.72
One factor (11 items)	52.26^*^	44	0.98	0.97	0.00 (0.00–0.05)	0.10	0.72

* *p* < 0.05.

**Table 5:** Standardized CFA factor loadings of 12 items model and 11 items model (Study 2: CFA, *N* = 178).

Items	Study 2 CFA loadings 12 items model	Study 2 CFA loadings 11 items model
Item 18	0.78	0.79
Item 25	0.76	0.76
Item 11	0.74	0.75
Item 15	0.63	0.63
Item 16	0.60	0.60
Item 10	0.42	0.37
Item 9	0.48	0.48
Item 13	0.69	0.63
Item 5	0.35	0.34
Item 28	0.45	0.45
Item 4	0.28	0.28
Item 23	0.23	Excluded

#### The concurrent validity of Bangla Rotter’s I-E scale

Table 6 summarizes the correlation coefficients among the total score of ICI (Duttweiler, 1984), neuroticism, openness to experience (Muhammad et al., 2011) and Bangla Rotter’s I-E scale (11 items). All of the correlations were statistically significant and supported our prediction of the direction of the relationships. Bangla Rotter’s I-E scale (11 items) was significantly positively correlated with neuroticism, *r* = 0.21, *p* < 0.01. Such a significant positive correlation was also reported by Horner (Horner, 1996), *r* = 0.33, *p* < 0.001. Internal control index (ICI) showed a significant negative correlation, *r* = −0.22, *p* < 0.01. Duttweiler (Duttweiler, 1984) also reported such correlation, *r* = −0.39, *p* < 0.001 between the ICI and “personal control” factor of Mirels (Mirels, 1970). Openness to experience also showed a significant negative correlation with Bangla Rotter’s I-E scale, *r* = −0.22, *p* < 0.01. Neville and Megha (2018) also reported such a significant negative correlation between LoC and openness to experience (*r* = −0.22, *p* < 0.01).

**Table 6:** Concurrent validity of Bangla Rotter’s I-E scale (*N* = 178).

	Rotter’s I-E scale	Neuroticism	Openness
Rotter’s I-E scale	-		
Neuroticism	0.21^*^	-	
Openness	−0.22^*^	−0.25^**^	-
Internal Control Index	−0.22^*^	−0.42^**^	0.46^**^

* *p* < 0.01;

** *p* < 0.001.

#### Item response theory-based analysis

IRT complements the conventional classical test theory-based analysis by gathering information on item discrimination and item difficulty. We gathered evidence on item quality of the Bangla Rotter I-E scale (11 items) as well as item fit, person fit and model by fitting a two-parameter logistic model (2PL) (Rozeboom et al., 1969) to the combined EFA sample and CFA sample (*N* = 478) in RStudio with the “mirt” package (Chalmers, 2012). In the combined sample, 68% (326) of the participants were female, ranging in age from 21 to 53 (29.18 ± 4.93) and 32% (152) of the participants were male, ranging in age from 21 to 45 (32.77 ± 4.05). Seventy-four percent (354) of the participants were married. The average years of education for the males were 16.64 ± 0.80 and for the female were 15.23 ± 2.11.

To assess the sampling adequacy for IRT analysis, we did a Monte Carlo simulation using “SimDesign” package (Chalmers and Adkins, 2020) with sample sizes varying from 50 to 350 and calculated the average root mean squared error (RMSE) to estimate the optimal sample size for the 2PL model with 11 items. The RMSE became stable for *N* = 300–350. Our combined sample size was larger than the estimated sample size for stability.

It required 18 iterations (Log-likelihood - 2825.749) for the 2PL model to converge. Item fit statistics signed chi-square test (S-*χ*^2^) (Orlando and Thissen, 2000; Orlando and Thissen, 2003) indicated all items were a good fit (Table 7). Model fit statistics estimated from the model indicated a best fit for the 2PL model, *M*_2_ = 59.42, *df* = 44, *p* = 0.06, RMSEA = 0.03, CFI = 0.98, TLI = 0.98.

**Table 7:** Item discrimination, difficulty and item fit statistics of Bangla Rotter I-E Scale with 11 items (*N* = 478).

Items	Discrimination	Difficulty	S-*χ*^2*^	*df*	*p*
Item 18	2.21	−1.09	6.42	4.00	0.17
Item 25	1.64	−0.55	9.82	6.00	0.13
Item 11	1.80	−0.04	2.96	6.00	0.81
Item 15	1.41	−0.14	3.78	6.00	0.71
Item 16	1.47	0.78	2.52	6.00	0.87
Item 10	0.96	2.79	9.33	6.00	0.16
Item 09	0.95	0.98	4.24	7.00	0.75
Item 13	0.98	−0.16	5.21	7.00	0.63
Item 05	0.77	2.40	4.41	7.00	0.73
Item 28	0.87	1.55	1.04	7.00	0.99
Item 04	0.49	0.31	9.94	7.00	0.19

* S-*χ*^2^: Signed Chi-square.

**Figure 5:**
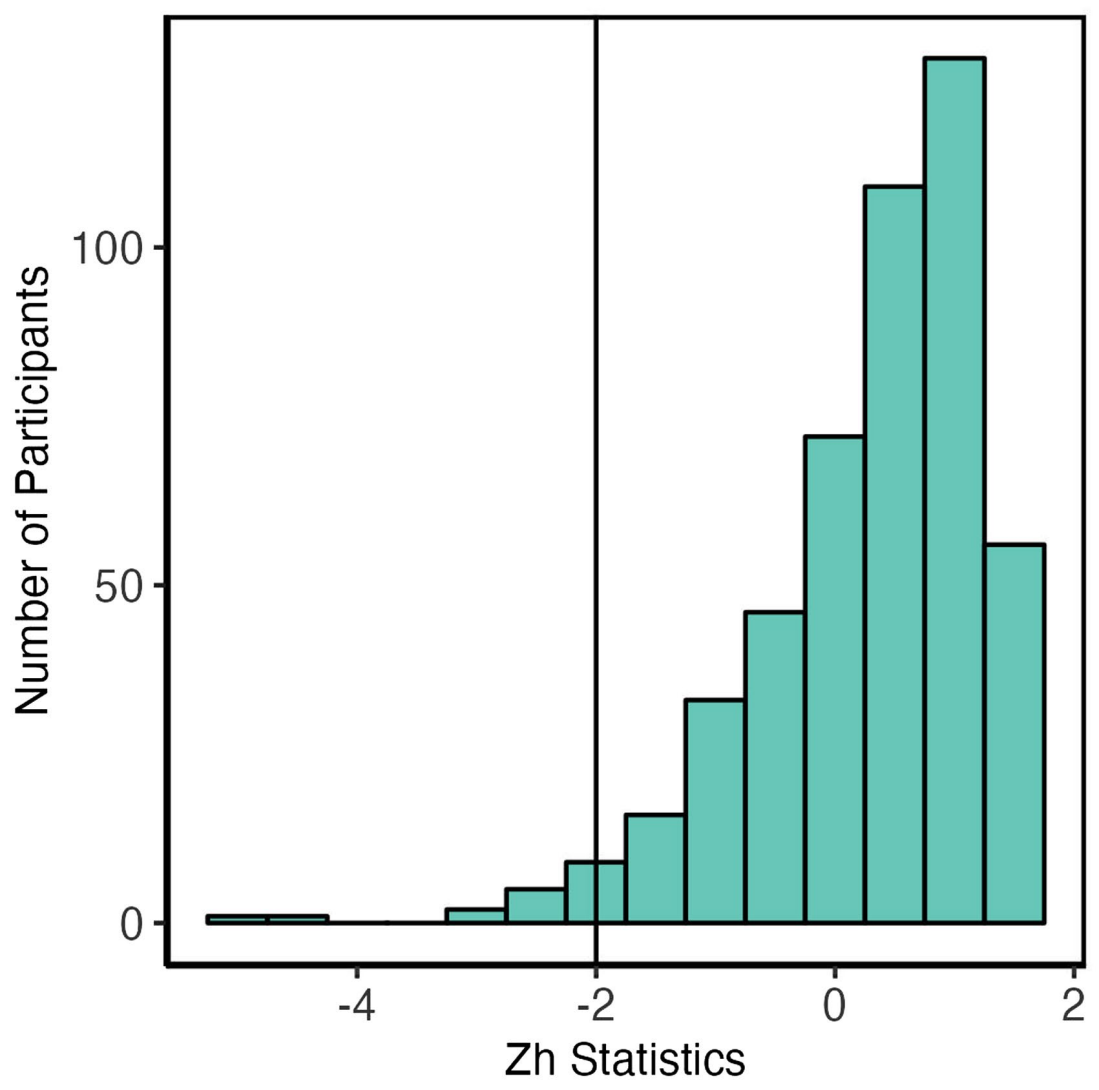
Person fit: Distribution of the *Z*_h_ statistic of the 2PL model. *Z*_h_ is larger than-2 for most participants indicating a good fit for the IRT model.

Person fit indicates the validity and meaningfulness of the fitted model at the participants’ latent trait level (Embretson and Reise, 2000). We estimated the person fit statistics using standardized fit index Z_h_ statistics (Drasgow et al., 1985). *Z*_h_ < −2 should be considered a misfit. Figure 5 indicates that *Z*_h_ is larger than-2 for most participants, suggesting a good fit for the selected IRT model.

Among the 11 items summarized in Table 7, one item (item 04) had low discrimination, five items showed moderate discrimination (items 05, 09, 10, 13, 28), three items high discrimination (items 15, 16 and 25) and two items (items 11 and 18) had very high discrimination capability. Item discrimination values ranged from 0.49 to 2.21. All items except items 18 and 04 were in the suggested guidelines of item discrimination parameter: 0.5≤ item discrimination ≥2.0 (Baker and Kim, 2017).

**Figure 6:**
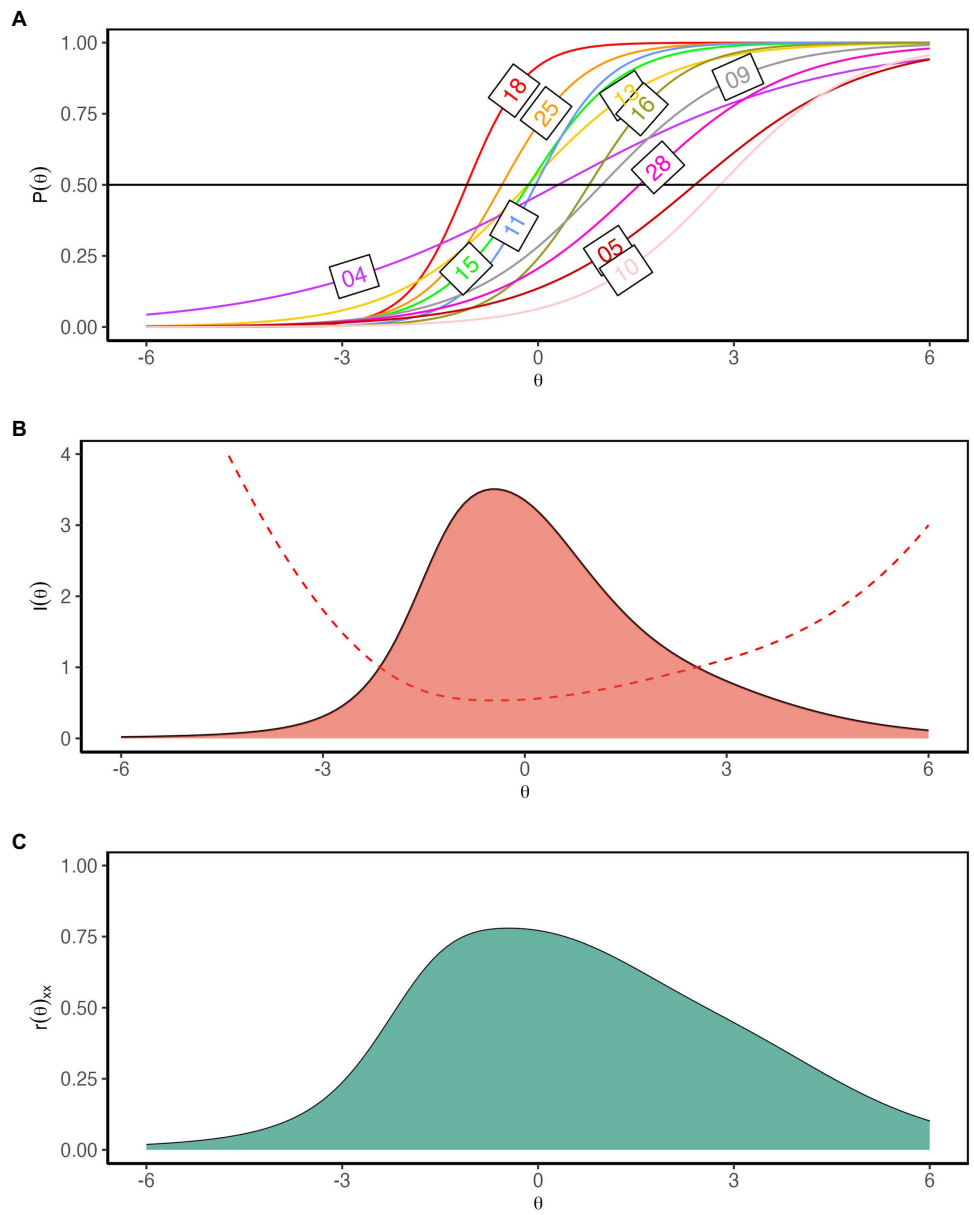
**(A)** Item characteristic curves (ICC) of the 11 items of the Bangla Rotter I-E scale. ICC indicates Bangla Rotter I-E scale is composed of easy (18 and 25), medium (4, 11, 13, 15), hard (9, 16, 28) and very hard items (5 and 10). **(B)** Test information curve. The curve’s peak is centered between the *θ* range −1–0.3. **(C)** Reliability plot. The scale’s reliability was 0.70 and above between *θ* = −1.4 and *θ* = 0.9.

The relationship between participants’ latent traits and the probability of responding to the preferred response option for the items is shown by the item characteristics curve (ICC; Figure 6A). An examination of the ICCs reveals that our items in the Bangla Rotter’s I-E scale covered a sizable range of underlying locus of control trait. The scale has two easy items (items 18 and 25), four medium difficulty items (items 4, 11, 13, and 15), three hard items (items 9, 16, and 28) and two very hard items (Items 5 and 10). The item difficulty ranged from −1.09 to 2.79 indicating all items were within the suggested guideline: −3.0≤ item difficulty ≤3.0 (Baker and Kim, 2017).

Figure 6B showed the measurement precision of the Bangla Rotter’s I-E scale as depicted by the standard error of estimated IRT scores for the 11 items (dashed line) with the test information curve (TIC) of the scale. TIC indicates the amount of information full-scale carries along the latent trait continuum. Standard errors of estimation provided information on the precision of the test in measuring the ability. Figure 6B indicated that Bangla Rotter’s I-E scale had the least standard error of measurement and carried the highest level of information between *θ* = −1 and *θ* = 0.3 with a peak at *θ* = −0.5. Figure 6C depicted the reliability estimates across the latent trait continuum and indicated the scale’s reliability was 0.70 and above between *θ* = −1.4 and *θ* = 0.9. The amount of information changed steadily with the change of *θ* across the continuum. The marginal reliability of the scale was 0.72 indicating the test’s overall satisfactory reliability (Green et al., 1984; Reise and Revicki, 2015).

**Figure 7:**
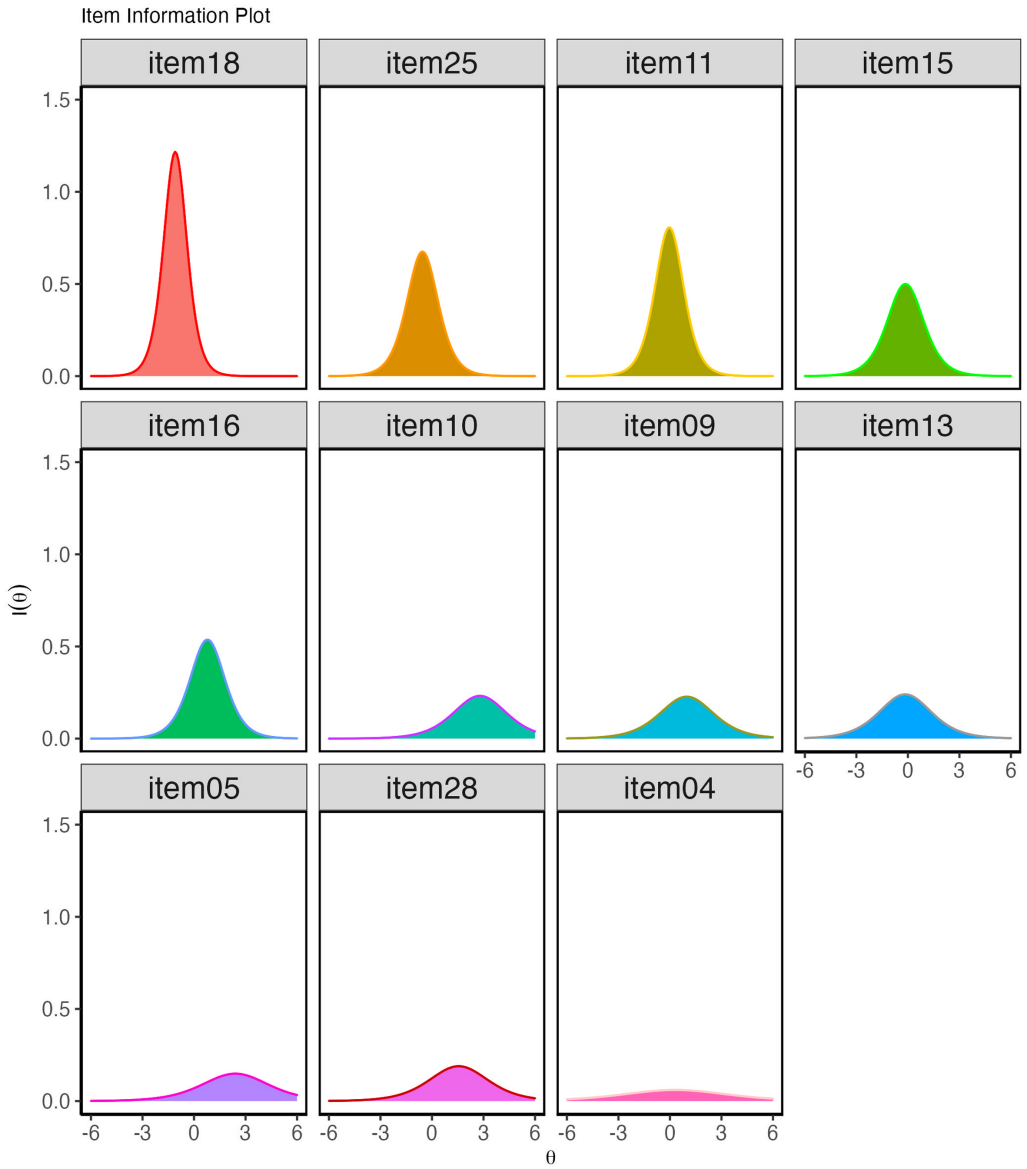
Item Information curves of Bangla Rotter’s I-E scale. Item 18 carried the highest level of information across the theta continuum, and item 04 carried the lowest information.

Item information curve (IIC) indicates the amount of information an item carries along the latent trait continuum. An examination of the IICs (Figure 7) revealed that item 18 had the highest information between *θ* levels −2 to 1. Item 4 was less informative with almost flat IIC along the trait continuum. Items 5, 9, 10, 13, and 28 only carried a little bump of information on the right-hand side of the theta continuum. Item information depends on item discrimination, where items with higher discrimination power provide higher information. The aforementioned six items’ had a low to moderate item discrimination index, thus providing low information. The rest of the items carried sufficient information across the theta continuum. Items 11, 13, 15, 16, and 25 had information peaks roughly centered on the measured trait (*θ*). Items 5, 9, 10, and 28 had information peaks on the external LoC area.

Considering the reliability estimates, standard error of measurement and test information curve, we conferred that the LoC was estimated with precision near the center of the trait continuum with a peak in the ranges of *θ* = −1 and *θ* = 0.3, which is sufficient to discriminate between external LoC and internal LoC (Baker and Kim, 2017). This adequacy is reflected by the correlation coefficient of the estimated θ and the obtained score in the Rotter’s I-E scale, *r* = 0.98, *p* < 0.001.

## General discussion

We followed the ITC (Bartram et al., 2018) guidelines to culturally adapt Rotter’s I-E scale into Bangla and psychometrically evaluate it by gathering evidence of validity (content, structural, and concurrent) (Furr, 2014) and reliability (internal consistency). We also gathered information about item quality using IRT.

We started with the initial 23 (excluding the six filler items) pairs of original items and translated them into Bangla following the standard forward-backward translation procedure (Study 1). The content validity of the initial synthesized scale was assessed by calculating I-CVI and S-CVI (average) (Lynn, 1986; Polit et al., 2007) from the evaluation of eight mental health experts. The final I-CVI scores for each item were higher than 0.83 and S-CVI was 0.94, indicating adequate content validity (Lynn, 1986; Polit et al., 2007). We administered the Bangla Rotter’s I-E scale to a large sample (300) to explore its latent structure. In exploratory factor analysis, we obtained two solutions: a one-factor solution with 12 items and a two-factor solution with 14 items. The first factor of the two-factor solution and the one-factor solution both contained items stemming from the beliefs regarding personal control over desired goal attainment. The emergence of such a factor is in line with the previous research (Mirels, 1970; Tobacyk, 1978).

Mirels (Mirels, 1970) conducted a factor analysis with 23 original items on the data obtained from 316 undergraduate students (157 Females) and reported two factors - personal control and political control. His “personal control” factor described the respondent’s preference to assign greater or lesser value to personal ability than to luck in realizing the desired goal. Each of these items included statements that would affirm the respondents’ disposition on their fate vs. their ability and hard work (e.g., In the long run, people get the respect they deserve in this world/Unfortunately, an individual’s worth often passes unrecognized no matter how hard he tries). The second factor reported by Mirels (Mirels, 1970) focused on respondents’ beliefs regarding control over political events. Tobacyk (Tobacyk, 1978) also reported a similar two-factor solution for the full-scale administered to 199 Polish university students. In our two-factor model, the first factor resembled the “personal factor” discussed earlier but showed poor reliability estimates (McDonald’s *ω*_t_ = 0.63). However, our sample did not observe the “political control” factor. Our second factor contained five items stemming from beliefs on interpersonal relationships and luck. But this factor was less interpretable in terms of a common theme and showed a poor reliability estimate (McDonald’s *ω*_t_ = 0.39).

Our one-factor model contained all items of “personal control” reported by Mirels (Mirels, 1970) and focused on respondents’ beliefs regarding “personal control” over event’s outcome. Also, this one-factor solution yielded satisfactory reliability estimate (McDonald’s *ω*_t_ = 0.70). Thus, we accepted the one-factor solution with 12 items. Such substantial reduction of item numbers was also observed in Niles (Niles, 1981) work. He examined the factor structure of Rotter’s I-E scale on adolescents sample of Sri Lanka (*N* = 192) and retained only 16 items. Tobacyk (Tobacyk, 1978) examined the factor structure among Polish university students (*N* = 199) and retained only 11 items.

A CFA on a separate sample (Study 2) gave a one-factor solution (CFI = 0.98, TLI = 0.97, RMSEA = 0.00) after discarding one item. The internal consistency McDonald’s *ω*_t_ of the 11-item scale was satisfactory (McDonald’s *ω*_t_ = 0.72) (Nájera Catalán, 2019). We gathered concurrent validity evidence by estimating correlations between the 11-item scale and neuroticism, openness to experience (Muhammad et al., 2011) and internal control index (Duttweiler, 1984). The ICI (Duttweiler, 1984) measures the same construct, LoC, a high score would indicate the internal LoC. On our scale, a high score would indicate an external LoC. Thus, a negative correlation is expected. Our scale showed a significant negative yet low correlation (*r* = −0.22, *p* < 0.01). Duttweiler (Duttweiler, 1984) also reported a moderate negative correlation between ICI and Mirels (Mirels, 1970) “personal control’ factor. They attributed the cause of such moderate correlation to the limited focus of the items in the ‘personal control’ factor. Like Mirels (Mirels, 1970), items retained in our scale limit their focus to the person’s disposition on luck or personal ability to attain the desired goal. In contrast, ICI encompasses items that also focus on self-image and willingness to act. As a result, such a correlation is expected.

LoC is believed to be correlated with behaviors and emotions related to neuroticism, such as maladaptive coping strategies (Taylor, 1983) and depression (Benassi et al., 1988). Previous studies reported that external LoC positively correlates with neuroticism (Morelli et al., 1979; Horner, 1996). Bangla Rotter’s I-E scale also showed a significant positive correlation with neuroticism (*r* = 0.21, *p* < 0.01). Literature also suggests the externals would score low on openness to experience (Sherman et al., 1973). Bangla Rotter’s I-E scale showed a significant negative correlation with openness to experience, *r* = −0.22, *p* < 0.001. From this evidence, we conferred that our adapted scale has satisfactory concurrent validity.

Lastly, we gathered information on the quality of items retained in our scale by IRT. We fitted a two-parameter logistic model (2PL) to our data. The fit indices indicated a best fit of the model, (*M*2 = 59.42, *df* = 44, *p* = 0.06, RMSEA = 0.03 [0.00–0.04], CFI = 0.98, TLI = 0.98). No item was identified as a misfit item. In terms of item difficulty, our scale contained easy, medium, hard and very hard items and covered a substantial range of underlying LoC attributes. Additionally, all items (except items 18 & 04) were also exhibiting item discrimination within the suggested range (Baker and Kim, 2017). Test information curve also indicated adequate ability to discriminate between external LoC and internal LoC with precision as the peak of the curve centered near the center of the continuum at *θ* = −1 and *θ* = 0.3. Also, the high correlation between the estimated *θ* score and the obtained score (*r* = 0.98, *p* < 0.001) in our scale indicated the efficiency of our adapted scale. However, in an ideal scenario, the test information curve should be zero-centered. In our scale, the peak of the test information curve is located slightly left of the center.

### Future direction

We explored the latent structure of Bangla Rotter’s I-E scale using a nonrandomized sample, and we did not find any factor related to political control. The overrepresentation of female participants in the current study is likely to account for this outcome, as they are less encouraged to give their voice in political matters in Bangladesh. Further studies should recruit a randomized gender-balanced sample to testify to this claim. Also, in IRT analysis, the test information curve should be zero-centered. However, our scale’s test information curve centered slightly left to zero. Future studies may investigate if this could be improved by adding items to the scale with an information peak on the center’s right side.

## Conclusion

The Bangla-translated Rotter’s I-E scale gave a one-factor solution with 12 items in exploratory factor analysis. A confirmatory factor analysis with this 12-item scale again offered a one-factor solution, but one more item was discarded this time. The scale appeared reliable (internal consistency) and valid (content, structural, and concurrent validity). IRT analysis on this scale revealed that this scale was composed of items covering a good range of underlying locus of control with diverse slope parameters, indicating a good range of discrimination. Hence, we recommend that Bangla Rotter’s I-E be used to measure the LoC of Bangla-speaking people, thus facilitating the understanding of different behavior domains related to locus of control, including academic success, health-related activities, professional competence and consumer behaviors (Findley and Cooper, 1983; Foon, 1987; Mantesso et al., 2008; Jacobs-Lawson et al., 2011; Karaman et al., 2018).

## Data availability statement

All data, analysis code underlying this article and an R-markdown reproducible manuscript have been made publicly available on GitHub and can be accessed at https://github.com/mind-psychometry/Bangla-Rotter-I-E-Scale.

## Ethics statement

The studies involving human participants were reviewed and approved by Monash University Human Research Ethics Committee (Project ID: 30638). The patients/participants provided their written informed consent to participate in this study.

## Author contributions

MS and SH: conceptualization. MS: data curation, formal analysis, and writing–original draft. SH: supervision and writing–review and editing. All authors contributed to the article and approved the submitted version.

## Conflict of interest

The authors declare that the research was conducted in the absence of any commercial or financial relationships that could be construed as a potential conflict of interest.

## Publisher’s note

All claims expressed in this article are solely those of the authors and do not necessarily represent those of their affiliated organizations, or those of the publisher, the editors and the reviewers. Any product that may be evaluated in this article, or claim that may be made by its manufacturer, is not guaranteed or endorsed by the publisher.

## Supplementary material

The Supplementary material for this article can be found online at: https://www.frontiersin.org/articles/10.3389/fpsyg.2022.1023856/full#supplementary-material

## References

[ref1] Andriessen J. Van Cadsand J. (1983). An analysis of the Dutch IE scale. Ned. Tijdschr. Psychol. 38, 7–24.

[ref2] Baker E. K. (1979). The relationship between locus of control and psychotherapy: a review of the literature. Psychother. Theory Res. Pract. 16, 351–362. doi: 10.1037/h0085901

[ref3] Baker F. B. Kim S. H. (2017). The Basics of Item Response Theory Using R. (New York: Springer), 27–34.

[ref4] Bandalos D. Finney S. (2019). Factor analysis: Exploratory and In The reviewer’s guide to quantitative methods in the social sciences, (pp. 98–122). Routledge. doi: 10.4324/9781315755649-8

[ref5] Barnier J. Briatte Z. Larmarange J. (2022). Questionr: Functions to Make Surveys Processing Easier. R package version 0.7.6. https://CRAN.R-project.org/package=questionr

[ref6] Bartlett M. (1954). A note on the multiplying factors for various chi-square approximations. J. R. Stat. Soc. B. Methodol. 16, 296–298.

[ref7] Bartram D. Berberoglu G. Grégoire J. Hambleton R. Muniz J. van de Vijver F. (2018). ITC guidelines for translating and adapting Tests (second edition). Int. J. Test. 18, 101–134. doi: 10.1080/15305058.2017.1398166

[ref8] Benassi V. A. Sweeney P. D. Dufour C. L. (1988). Is there a relation between locus of control orientation and depression? J. Abnorm. Psychol. 97, 357–367. doi: 10.1037/0021-843X.97.3.357, PMID: 3057032

[ref9] Bentler P. M. Chou C.-P. (1987). Practical issues in structural modeling. Sociol. Methods Res. 16, 78–117. doi: 10.1177/0049124187016001004

[ref10] Brown T. A. (2015). Confirmatory Factor Analysis for Applied Research. 2nd Edn. New York; London: The Guilford Press.

[ref11] Cattell R. B. (1966). The scree test for the number of factors. Multivar. Behav. Res. 1, 245–276. 10.1207/s15327906mbr0102_10 26828106

[ref12] Cavaiola A. A. Strohmetz D. B. (2009). Perception of risk for subsequent drinking and driving related offenses and locus of control among first-time DUI offenders. Alcohol. Treat. Q. 28, 52–62. doi: 10.1080/07347320903436169

[ref13] Chalmers R. (2012). Mirt: a multidimensional item response theory package for the R environment. J. Stat. Softw. 48, 1–29. doi: 10.18637/jss.v048.i06

[ref14] Chalmers R. Adkins M. (2020). Writing effective and reliable Monte Carlo simulations with the SimDesign package 16, 248–280. doi: 10.20982/tqmp.16.4.p248,

[ref15] Child D. (2006). Essentials of Factor Analysis. 3rd Edn. New York: Continuum.

[ref16] Comrey A. L. Lee H. B. (1992). A First Course in Factor Analysis, 2nd Edn. Hillsdale, NJ, US: Lawrence Erlbaum Associates Inc, xii–430.

[ref17] Costa P. T. McCrae R. R. (1992). Normal personality assessment in clinical practice: the NEO personality inventory. Psychol. Assess. 4, 5–13. doi: 10.1037/1040-3590.4.1.5

[ref18] Delsignore A. Schnyder U. (2007). Control expectancies as predictors of psychotherapy outcome: a systematic review. Br. J. Clin. Psychol. 46, 467–483. doi: 10.1348/014466507X226953, PMID: 17659158

[ref19] DeVellis R. F. (2006). Classical test theory. Med. Care 44, S50–S59. doi: 10.1097/01.mlr.0000245426.10853.30 17060836

[ref20] Diotaiuti P. Valente G. Mancone S. Falese L. Bellizzi F. Anastasi D.. (2021). Perception of risk, self-efficacy and social trust during the diffusion of Covid-19 in Italy. Int. J. Environ. Res. Public Health 18:3427. doi: 10.3390/ijerph18073427, PMID: 33806194 PMC8036340

[ref21] Drasgow F. Levine M. V. Williams E. A. (1985). Appropriateness measurement with polychotomous item response models and standardized indices. Br. J. Mathemat. Stat. Psychol. 38, 67–86. doi: 10.1111/j.2044-8317.1985.tb00817.x

[ref22] Duttweiler P. C. (1984). The internal control index: a newly developed measure of locus of control. Educ. Psychol. Meas. 44, 209–221. doi: 10.1177/0013164484442004

[ref23] Embretson S. E. Reise S. P. (2000). Item Response Theory for Psychologists. Mahwah, NJ, US: Lawrence Erlbaum Associates Publishers. xi–371.

[ref24] Fabrigar L. R. Wegener D. T. MacCallum R. C. Strahan E. J. (1999). Evaluating the use of exploratory factor analysis in psychological research. Psychol. Methods 4, 272–299. doi: 10.1037/1082-989X.4.3.272

[ref25] Findley M. J. Cooper H. M. (1983). Locus of control and academic achievement: a literature review. J. Pers. Soc. Psychol. 44, 419–427. doi: 10.1037/0022-3514.44.2.419

[ref26] Foon A. E. (1987). Locus of control as a predictor of outcome of psychotherapy. Br. J. Med. Psychol. 60, 99–107. doi: 10.1111/j.2044-8341.1987.tb02719.x 3304397

[ref27] Franklin R. D. (1963). YOUTH'S expectancies about internal versus external control of reinforcement related to N variables. Purdue University, Indiana, 47907.

[ref28] Furr R. M. (2014). Psychometrics: An Introduction. 2nd Edn. Thousand Oaks: SAGE.

[ref29] Garrido L. E. Abad F. J. Ponsoda V. (2013). A new look at Horn's parallel analysis with ordinal Variables. Psychol. Methods 18, 454–474. doi: 10.1037/a0030005, PMID: 23046000

[ref30] Graham J. M. (2006). Congeneric and (essentially) tau-equivalent estimates of score reliability: what they are and how to use them. Educ. Psychol. Meas. 66, 930–944. doi: 10.1177/0013164406288165

[ref31] Green B. F. Bock R. D. Humphreys L. G. Linn R. L. Reckase M. D. (1984). Technical guidelines for ASSESSING computerized adaptive TESTS. J. Educ. Meas. 21, 347–360. doi: 10.1111/j.1745-3984.1984.tb01039.x

[ref32] Hambleton R. K. Swaminathan H. Rogers H. J. (1991). Fundamentals of Item Response Theory (Measurement Methods for the Social Science). Vol. 2. Newbury Park: Sage Publictions, Inc.

[ref33] Hofstede G. (1980). Culture's Consequences: International Differences in Work-Related Values: Beverly Hills, California: Sage Publications.

[ref34] Horn J. L. (1965). A rationale and test for the number of factors in factor analysis. Psychometrika 30, 179–185. doi: 10.1007/BF02289447 14306381

[ref35] Horner K. L. (1996). Locus of control, neuroticism, and stressors: combined influences on reported physical illness. Personal. Individ. Differ. 21, 195–204. doi: 10.1016/0191-8869(96)00067-0

[ref36] Hutcheson G. D. (1999). The Multivariate Social Scientist: Introductory Statistics Using Generalized Linear Models. London: SAGE.

[ref37] Jackson D. L. (2003). Revisiting sample size and number of parameter estimates: some support for the N: q hypothesis. Struct. Equ. Model. 10, 128–141. doi: 10.1207/S15328007SEM1001_6

[ref38] Jacobs-Lawson J. M. Waddell E. L. Webb A. K. (2011). Predictors of health locus of control in older adults. Curr. Psychol. 30, 173–183. doi: 10.1007/s12144-011-9108-z

[ref39] Joe V. C. Jahn J. C. (1973). Factor structure of the rotter I-E scale. J. Clin. Psychol. 29, 66–68. doi: 10.1002/1097-4679(197301)29:1<66::AID-JCLP2270290125>3.0.CO;2-G

[ref40] John O. P. Donahue E. M. Kentle R. L. (1991). The Big Five Inventory--Versions 4a and 5b. Berkeley, CA: University of California,Berkeley, Institute of Personality and Social Research.

[ref41] John O. P. Naumann L. P. Soto C. J. (2008). “Paradigm shift to the integrative big five trait taxonomy: History, measurement, and conceptual issues,” in Handbook of Personality: Theory and Research. eds. John O. P. Robins R. W. Pervin L. A. (New York, London: The Guilford Press), 114–158.

[ref42] Kaiser H. F. (1974). An index of factorial simplicity. Psychometrika 39, 31–36. doi: 10.1007/BF02291575

[ref43] Karaman M. A. Nelson K. M. Cavazos V. J. (2018). The mediation effects of achievement motivation and locus of control between academic stress and life satisfaction in undergraduate students. Br. J. Guid. Couns. 46, 375–384. doi: 10.1080/03069885.2017.1346233

[ref44] Kazemi A. Kajonius P. (2021). Assessing person-centred care: an item response theory approach. Int. J. Older People Nursing 16:e12352-n/a. doi: 10.1111/opn.12352, PMID: 33111487

[ref45] Kline R. B. (2015). Principles and Practice of Structural Equation Modeling. New York; London: The Guilford Press.

[ref46] Kobasa S. C. Maddi S. R. Kahn S. (1982). Hardiness and health: a prospective study. J. Pers. Soc. Psychol. 42, 168–177. doi: 10.1037/0022-3514.42.1.168 7057354

[ref47] Kurtovic A. Vukovic I. Gajic M. (2018). The effect of locus of control on university Students' mental health: possible mediation through self-esteem and coping. J. Psychol. 152, 341–357. doi: 10.1080/00223980.2018.1463962, PMID: 30089081

[ref48] Lee H.-C. Chang C.-T. Cheng Z.-H. Chen Y.-T. (2018). Will an organic label always increase food consumption? It depends on food type and consumer differences in health locus of control. Food Qual. Prefer. 63, 88–96. doi: 10.1016/j.foodqual.2017.08.002

[ref49] Loffredo D. A. (1998). The relationships among ego states, locus of control, and dogmatism. Trans. Anal. J. 28, 171–173. doi: 10.1177/036215379802800211

[ref50] Lorenzo-Seva U. Timmerman M. Kiers H. (2011). The Hull method for selecting the number of common factors. Multivar. Behav. Res. 46, 340–364. doi: 10.1080/00273171.2011.564527, PMID: 26741331

[ref51] Lt H. Bentler P. M. (1999). Cutoff criteria for fit indexes in covariance structure analysis: conventional criteria versus new alternatives. Struct. Equ. Model. Multidiscip. J. 6, 1–55. doi: 10.1080/10705519909540118

[ref52] Lynn M. R. (1986). Determination and quantification of content validity. Nurs. Res. 35, 382–385. doi: 10.1097/00006199-198611000-00017, PMID: 3640358

[ref53] Mantesso J. Petrucka P. Bassendowski S. (2008). Continuing professional competence: peer feedback success from determination of nurse locus of control. J. Contin. Educ. Nurs. 39, 200–205. doi: 10.3928/00220124-20080501-02, PMID: 18512580

[ref54] Mardia K. V. (1970). Measures of multivariate skewness and kurtosis with applications. Biometrika 57, 519–530. doi: 10.1093/biomet/57.3.519

[ref55] Marsh H. W. Richards G. E. (1987). The multidimensionality of the Rotter I-E scale and its higher-order structure: an application of confirmatory factor analysis. Multivariate Behav Res. 22, 39–69. doi: 10.1207/s15327906mbr2201_3, PMID: 26811009

[ref56] Mirels H. L. (1970). Dimensions of internal versus external control. J. Consult. Clin. Psychol. 34, 226–228. doi: 10.1037/h0029005 5487591

[ref57] Morelli G. Krotinger H. Moore S. (1979). Neuroticism and Levenson's locus of control scale. Psychol. Rep. 44, 153–154. doi: 10.2466/pr0.1979.44.1.153, PMID: 461604

[ref58] Muhammad N Akter S Uddin E. (2011). Adaptation of Big Five Personality Test for Use in Bangladesh. Unpublished Manuscript. Jagannath University, Dhaka.

[ref59] Mulaik S. A. (2009). Foundations of Factor Analysis. London: Chapman and Hall/CRC.

[ref60] Nagelschmidt A. M. Jakob R. (1977). Dimensionality of Rotter's I-E scale in a Society in the Process of modernization. J. Cross-Cult. Psychol. 8, 101–112. doi: 10.1177/002202217781009

[ref61] Nájera Catalán H. E. (2019). Reliability, population classification and weighting in multidimensional poverty measurement: a Monte Carlo study. Soc. Indic. Res. 142, 887–910. doi: 10.1007/s11205-018-1950-z

[ref62] Neville R. Megha D. (2018). The relationship between self-actualization, locus of control and openness to experience. Indian J Posit. Psychol. 9, 238–241.

[ref63] Niles F. S. (1981). Dimensionality of Rotter's I-E scale in Sri Lanka. J. Cross-Cult. Psychol. 12, 473–479. doi: 10.1177/0022022181124007

[ref64] Novick M. R. Lewis C. (1967). Coefficient alpha and the reliability of composite measurements. Psychometrika 32, 1–13. doi: 10.1007/BF02289400, PMID: 5232569

[ref65] Orlando M. Thissen D. (2000). Likelihood-based item-fit indices for dichotomous item response theory models. Appl. Psychol. Meas. 24, 50–64. doi: 10.1177/01466216000241003

[ref66] Orlando M. Thissen D. (2003). Further investigation of the performance of S - X2: an item fit index for use with dichotomous item response theory models. Appl. Psychol. Meas. 27, 289–298. doi: 10.1177/0146621603027004004

[ref67] Polit D. F. Beck C. T. Owen S. V. (2007). Is the CVI an acceptable indicator of content validity? Appraisal and recommendations. Res. Nurs Health. 30, 459–467. doi: 10.1002/nur.20199, PMID: 17654487

[ref68] Pourhoseinzadeh M. Gheibizadeh M. Moradikalboland M. Cheraghian B. (2017). The relationship between health locus of control and health behaviors in emergency medicine personnel. Int J Community Based Nurs Midwifery 5, 397–407. 29043285 PMC5635559

[ref69] Reise S. P. Revicki D. A. (2015). Handbook of Item Response Theory Modeling: Applications to Typical Performance Assessment. London: Routledge.

[ref70] Revelle W. (2021). Psych: Procedures for Psychological, Psychometric, and Personality Research. Northwestern University, Evanston, Illinois, USA. Available at: https://CRAN.R-project.org/package=psych

[ref71] Rodriguez-Ricardo Y. Sicilia M. Lopez M. (2019). Altruism and internal locus of control as determinants of the intention to participate in crowdfunding: the mediating role of trust. J. Theor. Appl. Electron. Commer. Res. 14, 1–16. doi: 10.4067/S0718-18762019000300102

[ref72] Rosseel Y. (2012). Lavaan: an R package for structural equation modeling. J. Stat. Softw. 48, 1–36. doi: 10.18637/jss.v048.i02

[ref73] Rotter J. B. (1954). Social Learning and Clinical Psychology. New York: Prentice-Hall, Inc.

[ref74] Rotter J. B. (1955). “The role of the psychological situation in determining the direction of human behavior,” in Nebraska Symposium on Motivation. ed. Jones M. R. (Nebraska: University of Nebraska Press), 245–269.

[ref75] Rotter J. B. (1960). Some implications of a social learning theory for the prediction of goal directed behavior from testing procedures. Psychol. Rev. 67, 301–316. doi: 10.1037/h0039601, PMID: 13743936

[ref76] Rotter J. B. (1966). Generalized expectancies for internal versus external control of reinforcement. Psychol. Monogr. 80, 1–28. doi: 10.1037/h0092976, PMID: 5340840

[ref77] Rotter J. B. Chance J. E. Phares E. J. (1972). Applications of a Social Learning Theory of Personality. New York: Holt, Rinehart and Winston.

[ref78] Rozeboom W. W. Lord F. M. Novick M. R. Birnbaum A. (1969). Statistical Theories of Mental Test Scores. American Educational Research Journal, 6, 112–116.

[ref79] Schönbrodt F. D. Perugini M. (2013). At what sample size do correlations stabilize? J. Res. Pers. 47, 609–612. doi: 10.1016/j.jrp.2013.05.009

[ref80] Schwartz S. H. (1990). Individualism-collectivism: critique and proposed refinements. J. Cross-Cult. Psychol. 21, 139–157. doi: 10.1177/0022022190212001

[ref81] Schwartz S. H. (1992). Universals in the content and structure of values: theoretical advances and empirical Tests in 20 countries. Adv. Exp. Soc. Psychol. 25, 1–65.

[ref82] Shapiro S. S. Wilk M. B. (1965). An analysis of variance test for normality (complete samples). Biometrika 52, 591–611. doi: 10.1093/biomet/52.3-4.591

[ref83] Sherman M. F. Pelletier R. J. Ryckman R. M. (1973). Replication of the relationship between dogmatism and locus of control. Psychol. Rep. 33, 749–750. doi: 10.2466/pr0.1973.33.3.749, PMID: 4767831

[ref84] Siraji M. A. (2021). Create Publication Quality Tables and Plots. R package version 0.0.3. https://CRAN.R-project.org/package=tabledown

[ref85] Smidt W. Kammermeyer G. Roux S. Theisen C. Weber C. (2018). Career success of preschool teachers in Germany - the significance of the big five personality traits, locus of control, and occupational self-efficacy. Early Child Dev. Care 188, 1340–1353. doi: 10.1080/03004430.2017.1314275

[ref86] Smith P. B. Trompenaars F. Dugan S. (1995). The Rotter locus of control scale in 43 countries: a test of cultural relativity. Int. J. Psychol. 30, 377–400. doi: 10.1080/00207599508246576

[ref87] Stevens J. (2009). Applied Multivariate Statistics for the Social Sciences. 5th Edn. New York, NY: Routledge.

[ref88] Taylor S. E. (1983). Adjustment to threatening events: a theory of cognitive adaptation. Am. Psychol. 38, 1161–1173. doi: 10.1037/0003-066X.38.11.1161

[ref89] Team R. C. (2021). R: A Language and Environment for Statistical Computing. R Foundation for Statistical Computing, Vienna, Austria. https://www.R-project.org/

[ref90] Tobacyk J. (1978). Factor structure of Rotter's I-E scale in female polish university students. J. Soc. Psychol. 106, 3–10. doi: 10.1080/00224545.1978.9924139, PMID: 28135528

[ref91] Tyler F. B. Dhawan N. Sinha Y. (1989). Cultural contributions to constructing locus-of-control attributions. Genet. Soc. Gen. Psychol. Monogr. 115, 205–220. 2767420

[ref92] Velicer W. (1976). Determining the number of components from the matrix of partial correlations. Psychometrika 41, 321–327. doi: 10.1007/BF02293557

[ref93] Watkins M. (2020). A Step-by-Step Guide to Exploratory Factor Analysis with R and RStudio. Routledge.

[ref94] Watson J. M. (1981). A note on the dimensionality of the rotter locus of control scale. Aust. J. Psychol. 33, 319–330. doi: 10.1080/00049538108254701

[ref95] Witt L. A. (1988). Locus of control and success as a professional money collector. J. Soc. Psychol. 128, 703–704. doi: 10.1080/00224545.1988.9922927

[ref96] Worthington R. L. Whittaker T. A. (2006). Scale development research: a content analysis and recommendations for best practices. Couns. Psychol. 34, 806–838. doi: 10.1177/0011000006288127

[ref97] Yu C.-Y. (2002). Evaluating cutoff criteria of model fit indices for latent variable models with binary and continuous outcomes. University of California, Los Angeles.

