# Supplementary material for: Psychometric evaluation of the Bangla-Translated Rotter’s Internal-External Scale through classical test theory and item response theory
Source: Front Psychol. 2022 Nov 11;13:1023856. doi: 10.3389/fpsyg.2022.1023856 (PMC9692010; doi:10.3389/fpsyg.2022.1023856)
Supplement: Supplementary file 1 [file Table_1.pdf]

Table S1. Inter-item tetrachoric correlation coefficients for the 23-item Rotter I-E Scale (Study 1; N = 300).

	item2	item3	item4	item5	item6	item7	item9	item10	item11	item12	item13	item15	item16	item17	item18	item20	item21	item22	item23	item25	item26	item28	item29
item2	1.00																						
item3	0.04	1.00																					
item4	0.09	0.00	1.00																				
item5	0.01	0.11	0.17	1.00																			
item6	0.14	0.09	0.11	-0.14	1.00																		
item7	0.30	0.19	-0.01	0.00	0.27	1.00																	
item9	0.34	0.12	-0.07	0.18	0.14	0.26	1.00																
item10	0.15	-0.22	0.18	0.29	0.11	0.07	0.28	1.00															
item11	0.09	0.04	0.23	0.37	-0.03	0.01	0.31	0.27	1.00														
item12	0.05	0.07	0.33	0.30	0.05	0.06	0.16	0.09	0.09	1.00													
item13	0.33	0.16	0.31	0.25	0.04	0.01	0.19	0.07	0.37	0.14	1.00												
item15	0.06	0.22	0.22	0.14	0.03	0.14	0.25	0.37	0.49	0.18	0.20	1.00											

	item2	item3	item4	item5	item6	item7	item9	item10	item11	item12	item13	item15	item16	item17	item18	item20	item21	item22	item23	item25	item26	item28	item29
item16	0.23	0.07	0.17	0.21	-0.03	0.07	0.25	0.27	0.53	0.07	0.25	0.47	1.00										
item17	0.03	0.17	-0.04	0.05	-0.17	0.13	0.13	-0.01	0.08	0.05	0.21	0.05	0.10	1.00									
item18	0.13	0.06	0.31	0.32	0.14	0.28	0.52	0.49	0.51	0.15	0.41	0.62	0.27	0.29	1.00								
item20	0.27	0.06	0.06	-0.05	-0.05	0.31	0.04	0.16	0.09	-0.03	0.18	0.16	0.08	0.06	0.08	1.00							
item21	0.14	0.09	-0.20	0.01	0.09	0.19	0.43	0.24	-0.03	0.17	0.01	0.22	0.07	0.25	0.15	0.27	1.00						
item22	-0.08	0.12	0.30	0.19	0.16	0.07	0.02	0.16	0.08	0.29	0.11	0.20	0.01	-0.03	0.25	0.02	0.14	1.00					
item23	0.44	0.07	0.20	0.42	0.16	0.01	0.11	0.41	0.26	0.36	0.34	0.24	0.41	-0.04	0.14	0.08	0.43	0.04	1.00				
item25	0.14	0.12	0.16	0.32	0.06	0.27	0.43	0.35	0.42	0.13	0.29	0.38	0.51	0.25	0.57	0.22	0.17	0.18	0.37	1.00			
item26	0.00	0.29	0.20	-0.11	-0.21	0.11	0.03	0.13	0.00	0.21	0.20	0.09	-0.02	0.23	0.06	0.33	0.11	0.12	0.05	0.23	1.00		
item28	0.09	-0.19	0.18	0.14	0.04	0.23	0.31	0.20	0.29	0.23	0.17	0.27	0.35	0.04	0.39	0.13	0.15	0.18	0.20	0.22	-0.07	1.00	
item29	0.07	0.08	0.04	0.09	-0.05	0.22	0.24	0.30	0.19	0.12	0.08	0.10	0.04	0.19	-0.02	-0.02	0.04	0.29	-0.02	0.13	0.13	0.10	1.00
