# Supplementary material for: Psychometric evaluation of the Bangla-Translated Rotter’s Internal-External Scale through classical test theory and item response theory
Source: Front Psychol. 2022 Nov 11;13:1023856. doi: 10.3389/fpsyg.2022.1023856 (PMC9692010; doi:10.3389/fpsyg.2022.1023856)
Supplement: Supplementary file 2 [file Table_2.pdf]

Table S2. Minimum average partial (MAP) factor extraction method (Study 1; N = 300). MAP Statistics is the lowest for the first factor, indicating that only one factor can sufficiently describe the latent structure.

Factor Number	MAP Statistic	df	χ^2	BIC
1	0.007	230.00	348.16*	-963.71
2	0.009	208.00	296.79*	-889.59
3	0.011	187.00	251.80*	-814.81
4	0.013	167.00	203.85*	-748.68
5	0.016	148.00	162.38*	-681.78
6	0.019	130.00	138.05*	-603.44
7	0.023	113.00	109.60*	-534.92
8	0.028	97.00	89.70*	-463.56

*p <.001

MAP Statistics and Bayesian Information Criterion (BIC) are lowest for factor number = 1.
