# Supplementary material for: Psychometric evaluation of the Bangla-Translated Rotter’s Internal-External Scale through classical test theory and item response theory
Source: Front Psychol. 2022 Nov 11;13:1023856. doi: 10.3389/fpsyg.2022.1023856 (PMC9692010; doi:10.3389/fpsyg.2022.1023856)
Supplement: Supplementary file 3 [file Data_Sheet_1.pdf]

File S1: Bangla Rotter’s Internal-External Scale

নির্দেশনা:

নিচে একজন ব্যক্তির জীবনে সমাজের বিভিন্ন ঘটনা কিভাবে প্রভাব বিস্তার করে তা অনুসন্ধাণের জন্য এগারোটি বাক্য-জোড়া আছে। প্রতিটি বাক্য-জোড়া থেকে আপনাকে যেকোন একটি বাক্য নির্বাচন করতে হবে। কিছু কিছু বাক্য-জোড়ার ক্ষেত্রে আপনার কাছে মনে হতে পারে যে প্রদত্ত দু’টি বাক্যই আপনার জন্য প্রযোজ্য অথবা কোনটিই প্রযোজ্য নয়। সেক্ষেত্রে আপনি প্রদত্ত বাক্য দু’টির মাঝে যেটি আপনার কাছে অধিক গ্রহণযোগ্য মনে হবে সেটি নির্বাচন করুন। । মনে রাখবেন, এখানে কোন সঠিক বা ভুল উত্তর নেই। আপনার সময়ের জন্য আন্তরিক ধন্যবাদ।

বাক্য-জোড়া	**বাক্য-ক**	**বাক্য-খ**
১	শেষ পর্যন্ত এই পৃথিবীতে মানুষ তার প্রাপ্য মর্যাদা পাবেই।	দূর্ভাগ্যবশত, ব্যক্তির সর্বোচ্চ চেষ্টা থাকার পরেও তার প্রকৃত মূল্য প্রায়শ নজরের আড়ালে থেকে যায়।
২	“শিক্ষকেরা ছাত্রদের সাথে ন্যায়বিচার করেন না”-এই ধারণাটি একটি ভ্রান্ত ধারণা।	অধিকাংশ ছাত্ররা আন্দাজও করতে পারবে না তাদের পরীক্ষা পত্রের নম্বর ভাগ্যের কতটা নির্ভর করে।
৩	আমি অনেক সময় দেখেছি যে, ভাগ্যে যা আছে তাই হয়।	কোন সিদ্ধান্ত গ্রহণে ভাগ্যের উপর বিশ্বাস করা কখনোই আমার জন্যে ভালো হয় নি।
৪	যেসব ছাত্ররা পরীক্ষার জন্য ভালভাবে প্রস্তুতি নেয় তাদের জন্য কোন পরীক্ষাই কঠিন নয়।	অনেক সময় কোর্সের সাথে সম্পর্কহীন এমন প্রশ্ন আসে যে প্রস্তুতি নেয়া পণ্ডশ্রম।
৫	পরিশ্রমের মাধ্যমে সফলতা আসে। ভাগ্যের আসলে তেমন কোন অবদান নেই।	একটি ভাল চাকরি অনেকাংশে নির্ভর করে সঠিক সময়ে সঠিক জায়গায় উপস্থিতির উপর।
৬	আমি যখন কোন কিছু পরিকল্পনা করি, আমি নিশ্চিত থাকি যে আমার পরিকল্পনা কাজ করবে।	অনেক আগে থেকে কোন কিছু পরিকল্পনা করা ঠিক নয় কারণ সময়ের সাথে ভাগ্য অনেক কিছু পরিবর্তন করতে পারে।
৭	আমার অর্জনের ক্ষেত্রে ভাগ্যের ভূমিকা নেই বললেই চলে।	অনেক সময় আমরা কি করবো তার সিদ্ধান্ত ভাগ্যের আপর ছেড়ে দেই
৮	কে অফিস প্রধান হবে তা অনেকাংশে নির্ভর করে সঠিক সময়ে সঠিক জায়গায় উপস্থিতির উপর।	সঠিক কাজ করা নির্ভর করে দক্ষতার উপর। এ ব্যাপারে ভাগ্যের কোন ভূমিকা নেই।
৯	ভাগ্য আমাদের জীবনকে কতটা নিয়ন্ত্রণ করতে পারে তা অনেকের ধারণার বাইরে।	ভাগ্য বলে কিছু নেই।
১০	আমার সাথে যা কিছু ঘটে তার উপর আমার নিয়ন্ত্রণ খুব কম।	আমার জীবনে ভাগ্যের ভূমিকা প্রধান-এটা বিশ্বাস করা আমার জন্য অসম্ভব।
১১	আমার জীবনে যা হবে তা আমার কর্মফল।	মাঝে মাঝে মনে হয় আমার জীবনের বৈঠা আমার হাতে নেই।

Psychometric Properties:

Bangla Rotter’s I-E scale has a one-factor latent structure. Internal consistency McDonald's omega total estimated on a Bangladeshi adult sample (N= 178) was .72.

Scoring:

Score one point for each of the following:

১.খ, ২.খ, ৩.ক, ৪.খ, ৫.খ, ৬.খ, ৭.খ, ৮.ক, ৯.ক, ১০.ক, ১১.খ

A high total score = External Locus of control

A low total score = Internal Locus of Control
